# Guided Bone Regeneration in a Periodontally Compromised Individual with Autogenous Tooth Bone Graft: A Radiomics Analysis

**DOI:** 10.3390/jfb14040220

**Published:** 2023-04-14

**Authors:** Jingyu Li, Feifan Jin, Renfei Wang, Xiaodan Shang, Peiran Yang, Yuchi Zhu, James K. H. Tsoi, Ki Chan, Shuhua Wang

**Affiliations:** 1 School/Hospital of Stomatology, Zhejiang Chinese Medical University, Hangzhou 310000, China; 202111125011014@zcmu.edu.cn (J.L.); 202111125011010@zcmu.edu.cn (F.J.); 201812211601003@zcmu.edu.cn (X.S.); 201912213604028@zcmu.edu.cn (P.Y.); 201912211602004@zcmu.edu.cn (Y.Z.); 2 Dental Materials Science, Applied Oral Sciences and Community Dental Care, Faculty of Dentistry, The University of Hong Kong, Hong Kong 999077, China; jkhtsoi@hku.hk; 3 Restorative Dental Sciences, Faculty of Dentistry, The University of Hong Kong, Hong Kong 999077, China; h0492142@hku.hk

**Keywords:** autogenous tooth bone graft material (AutoBT), socket preservation, severe periodontitis, artificial bone grafting, CBCT scans

## Abstract

Background: Autogenous tooth bone graft material (AutoBT) has been advocated as a bone substitute when conducting alveolar ridge preservation. This study is aimed at using a radiomics approach in order to evaluate and testify whether AutoBT can stimulate bone growth during socket preservation in severe periodontal cases. Materials and Methods: For this study, 25 cases with severe periodontal diseases were selected. The patients’ AutoBTs were inserted into the extraction sockets and covered with Bio-Gide^®^ collagen membranes. 3D CBCT scans and 2D X-rays were taken of the patients before surgery and after 6 months post-surgery. For the retrospective radiomics analysis, the maxillary and mandibular images were compared in different groups. Maxillary bone height was analyzed at the buccal, middle, and palatal crest sites, while the mandibular bone height was compared at the buccal, center, and lingual crest sites. Results: In the maxilla, the alveolar height was increased by −2.15 ± 2.90 mm at the buccal crest; −2.45 ± 2.36 mm at the center of the socket, and −1.62 ± 3.19 mm at the palatal crest, while the height of the buccal crest was increased by 0.19 ± 3.52 mm, and the height at the center of the socket was increased by −0.70 ± 2.71 mm in the mandible. The three-dimensional radiomics analysis demonstrated significant bone growth in the local alveolar height and high density. Conclusion: Based on clinical radiomics analysis, AutoBT could be used as an alternative bone material in socket preservation after tooth extraction in patients with severe periodontitis.

## 1. Introduction

Periodontitis is a serious infectious gingiva disease that causes alveolar bone absorption and soft tissue inflammation [1]. Studies have shown that not only can periodontitis severely impact a patient’s quality of life [2], but people with periodontal disease may also be at risk for other diseases such as Alzheimer’s disease [3,4,5,6], heart disease [7], diabetes [8], and rheumatoid arthritis [9]. For patients diagnosed with severe periodontitis (i.e., Periodontitis stage ≥ III) [10], it has been reported that their alveolar bone’s vertical absorption ranges between 11 and 22%, and their horizontal absorption ranges between 29 and 63% six months after tooth extraction [11]. In addition, the soft tissue collapses into the defect, preventing normal and natural healing [12]. It is also difficult to conduct an implantation procedure or dental prosthesis in a patient with severe bone loss. Several studies have shown various techniques that can increase jawbone volume in periodontal cases, including autogenous bone grafting [13], guided bone regeneration techniques [14], and maxillary sinus floor elevation [15]. F. Pourdanesh [16] suggested that a tenting approach can reduce large autogenous bone grafts in severely atrophic ridges and local bony defects, improving the longevity of the implant.

Autologous bone is an ideal material for the reconstruction of hard tissue defects with which to carry on bone augmentation because it promotes osteogenesis, osteoinduction, osteoconduction, and rapid healing. However, there are disadvantages of autologous bone as a graft material, such as limited harvest volume, inevitable resorption, and induction of a second defect in the donor area [17,18]. An organic deproteinized bovine bone mineral can be used as a scaffold for new bone formation, but it carries an indiscernible risk of prion disease transmission to patients and a lack of osteoinductivity [19]. Therefore, autogenous tooth bone graft material (AutoBT) has been developed as a bone replacement material [20].

AutoBT is an affordable, accessible, anti-infective, and low-immunogenicity bone replacement material [21,22,23]. As early as 1967, AutoBT was reported to release BMP-2, which could be an osteoinductive and bone-directed colloidal material [21,24]. In 2008, calcium and phosphorus were found in AutoBT particles, showing its potential to be used as a bone scaffold [17,18,25]. Recently, AutoBT was found to be an acid-soluble scaffold material, since it contains a collagen matrix, TGF-β, and G-CSF [22,23,26]. AutoBT, as well as reconstituted bone, exhibited less bone resorption compared to synthetic materials [23]. Since it is similar in composition to the autologous bone, consisting of 18% collagen, 2% non-collagenous proteins (NCPs), 70% hydroxyapatite (HA), and 10% body fluid (percentages indicating weight/volume) [27], it offers good biocompatibility and can help the patient overcome the possibility of bone rejection due to being made from biochemical materials [28]. Kim [29] performed bone grafting in 14 extraction sites and reported a 3-year implant survival rate of 92.85%. Jeong [30] used AutoBT to lift the maxillary sinus floor in 51 patients. A survival rate of 96.15% was achieved with 100 implants. During the procedure here, AutoBT is placed over the alveolar bone defect and covered with a Bio-Gide^®^ collagen membrane, which provides a space for the osteoprogenitor cells to colonize and crawl, acting as a guide for bone regeneration [31].

In the field of oral implantology, CBCT provides accurate three-dimensional images of the bone quality and volume of the jaws, as well as the tissue structure of the mandibular nerve canal and maxillary sinus [32]. CBCT is currently used to assess and analyze the volume, three-dimensional position, and elevation of the implant at the site preservation in order to determine whether a good clinical result has been achieved.

Chen [33] showed that oral panoramic film is still the major method of examination in primary care hospitals. Implant treatment can be carried out in patients with missing teeth in the posterior region, which has good clinical bone conditions according to the bone information measured by panoramic film observation. Moreover, oral panoramic film is often used to measure the height of the peri-implant alveolar bone and to assess the level of bone resorption at the implant margin [34].

CBCT still suffers from metallic artifacts that reduce image quality [35] and have a low resolution for soft tissue. At the same time, the imaging results of the digital curved body layer panoramic slice are relatively less disturbed by metal staples and crowns. When other imaging methods with lower radiation doses, such as curved body films, can meet the diagnostic and therapeutic needs of the operator, the corresponding imaging method should be used with the principle of minimizing the radiation dose, and the conditions of the use of CBCT should be strictly controlled. Thus, this study aims to evaluate AutoBT’s clinical effect on bone preservation in periodontitis patients by radiological analysis, in order to provide some reference for the effect of implant restoration in the future.

## 2. Materials and Methods

### 2.1. Ethics and Sample Selection

This study was conducted according to the Declaration of Helsinki principles. The retrospective study protocol was approved by the Medical Ethics Committee of Hangzhou Dental Hospital (23 January 2022). Written informed consent was obtained from each subject before participation in the study.

### 2.2. Patient Selection

This clinical patient selection was conducted from June 2018 to December 2020 at two hospitals: Hangzhou Stomatological Hospital and Hangzhou Zhongyi Stomatological Outpatient Department, with the following criteria:

(a) Patient inclusion criteria:
  1. No history of underlying disease;
  2. Extraction cases of single premolar or molar with no significant chronic endodontic lesions;
  3. Patients who have achieved or maintained a healthy periodontal status after ultrasonic scaling;
  4. Healthy oral hygiene: no obvious supragingival calculus, no obvious redness or swelling of the gingiva, Debris Index ≤ 1, Plaque index ≤ 1 after oral hygiene instruction and good compliance;
  5. Severe periodontal disease as defined [10] by alveolar bone resorption exceeding two-thirds of the root length of the tooth in X-ray, tooth mobility 2–3, probing depth > 6 mm.
(b) Patient exclusion criteria:
  1. Have severe systemic diseases and cannot undergo surgery;
  2. Have serious psychological or mental illness;
  3. Smoking more than 10 cigarettes per day;
  4. Pregnant and/or breastfeeding;
  5. Have an acute infection in the affected or adjacent teeth;
  6. Diagnosed with bone diseases, and have taken bisphosphonates for a long period;
  7. Patients with blood disorders, taking blood-thinning drugs.

**Figure 1:**
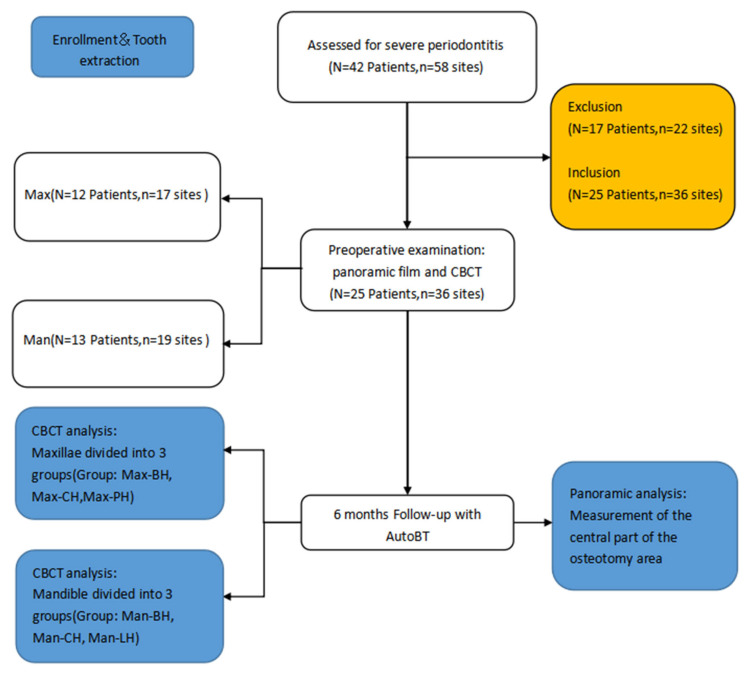
Flow diagram of the patient demographics and tooth extraction sites showcasing the study’s procedural methods.

Among the cases who visited the hospital, a total of 42 patients with 58 severe periodontitis sites were operated upon and qualified to be included in this study. Only 25 patients with 36 severe periodontitis sites were operated in the study (Figure 1).

### 2.3. Material Preparation

Equipment and materials:

1. BonMaker^®^ Automatic dental bone-graft-material preparation machine (Shenyang, China);
2. BonMaker^®^ Automatic dental bone-graft-material disinfection solution (Shenyang, China);
3. Geistlich Bio-Gide^®^ collagen membrane (Osteohealth, Zurich, Switzerland);
4. TaiHe^®^ Non-absorbent nylon suture (Chengdu, China)

#### AutoBT Preparation

**Figure 2:**
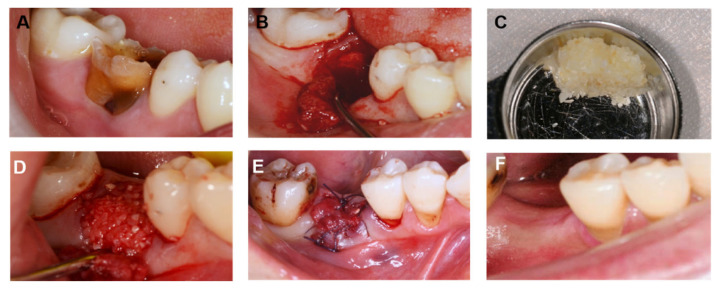
Photographs of a clinical case: (**A**) right mandibular first molar before extraction; (**B**) severe buccal bone defect and vertical release incision post-extraction; (**C**) mixture of AutoBT and the patient’s extracted tooth (particle size: 250 μm–1000 μm); (**D**) socket grafted with AutoBT; (**E**) graft site covered with a resorbable collagen membrane and non-absorbable sutures; (**F**) intraoral photograph of the clinical case 6 months post-surgery.

The teeth that had been removed from the patients due to periodontitis were selected. Surface soft tissues, including periodontal fibers and pulp, abrasional calculus, enamel, and cement were removed with a fast turbine. If caries were found, the healthy dentin was removed and dried with an air gun. A BonMaker^®^ Auto-Tooth Bone Graft System (BMK-AUTO-02) was used for grinding the tooth down and obtaining the boaxine powder used for treatment. The AutoBT dental-bone-powder disinfectant was poured into the device’s reagent tank and the AutoBT powder was obtained by the device (Figure 2C). About 1 mL of blood was removed from the patient’s alveolar socket and mixed with AutoBT powder.

### 2.4. Surgical Method

All patients received prophylactic antibiotic therapy (i.e., 250 mg of Cefuroxime, or 250 mg of Azithromycin if allergic to Penicillin) 30 min before tooth extraction, and an analgesic drug (i.e., 300 mg of Saridon) if needed and for as long as required. Patients rinsed with 0.2% chlorhexidine solution for 1 min before the procedure.

A submerged incision was made approximately 0.5 to 1 mm below the free gingival margin on the buccal and lingual sides of the tooth, and a reverse bevel incision was made on the bone crest in order to remove the lining of the periodontal pocket and to extract the affected tooth. The extraction socket was examined, and all granulation tissue was removed with a periosteal stripper and spatula. Thereafter, the wound was rinsed with sterile saline.

After evaluation, the extraction socket was filled with AutoBT (particle size: 250 to 1000 microns in diameter), and the (Bio-Gide^®^) collagen membrane was trimmed to the appropriate size and placed under the gingival margin 2.0~3.0 mm from the bone grafting granular margin. After relaxing the soft tissue flap, the wound was closed with interrupted sutures. After 1 week, the sutures were removed (Figure 2A–F).

All patients continued to take oral antibiotics three times per day for three consecutive days after surgery, and received injection treatment, if necessary, to manage postoperative discomfort and inflammation. Oral hygiene guidance and any necessary treatment related to periodontal health were provided throughout the study.

### 2.5. Observation Indicator

**Figure 3:**
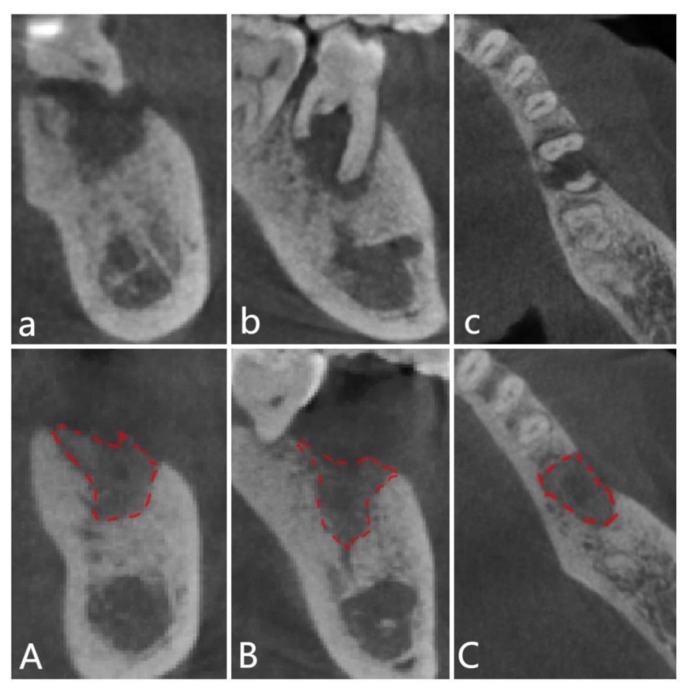
CBCT images of the (**a**,**A**) coronal; (**b**,**B**) sagittal; and (**c**,**C**) cross-sectional sections of the alveolar bone taken 6 months post-surgery. Red lines: Increased of the grafted site could be observed.

**Figure 4:**
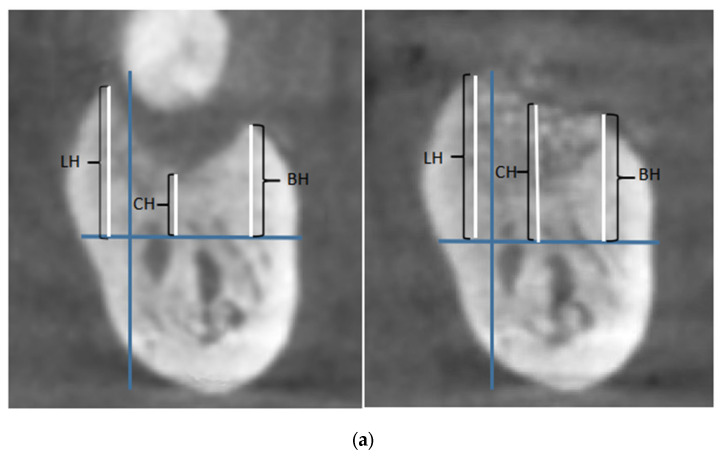

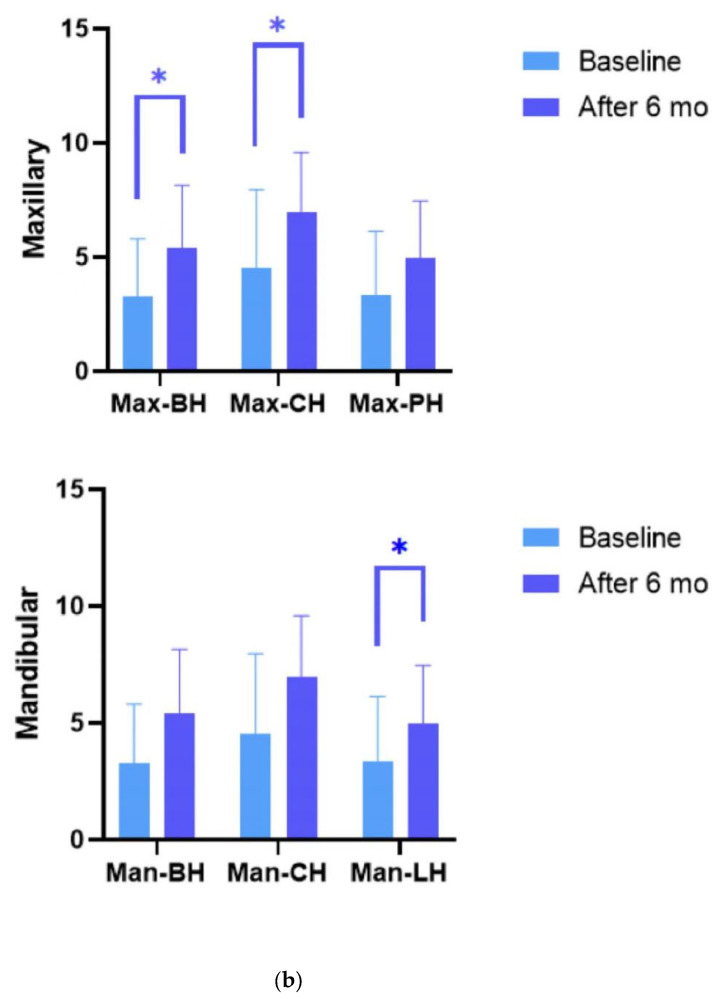
(**a**) Preoperative and postoperative comparison of the mandibular alveolar bone height. Table key: LH: height of the lingual crest; BH: height of the buccal crest; CH: height in the center of the socket; PH: height of the palatal crest. (**b**) Preoperative and postoperative comparison of the maxillary and mandibular alveolar bone height. Table key: BH: height of the buccal crest; CH: height in the center of the socket; LH: height of the lingual crest; PH: height of the palatal crest. * *p* < 0.05.

Cone-beam computer tomography (CBCT) (ORTHOPANTOMOGRAPH OP 3D, Prokavo, Tuusula, Finland) scan-rays were taken before surgery and 6 months post-surgery. Two sets of DICOM data were generated and transmitted to volume-imaging software (Mimics 19, Materialise), where 3D reconstruction and image analysis were conducted. Owing to differences in density and structure, the autogenous dentin material was easily distinguished from the residual bone. The lower margins of the mandible and the palatal dome of the maxilla were measured, and the virtual model was superimposed on the selected area of the data. After stacking, the two datasets were aligned and manually checked for a perfect match. The following imaging measurements were documented: the maxillary bone height at the buccal, middle, and palatal crest sites, and the mandibular bone height at the buccal, center, and lingual crest sites (Figure 3 and Figure 4a).

The Planmeca Romexis 3.8.2 software was used to display a curved body layer panorama with the enabled distance measurement function. The midpoint of the line between the enamel and bone borders of the adjacent teeth in the surgical area was set as A. If the implant was placed in the maxilla, the length of the AB line was obtained by intersecting the floor of the maxillary sinus at intersection B. The length of the AB line was obtained by intersecting the mandibular nerve canal at intersection B. If the implant was placed in the mandible, the length of the AB line was obtained by intersecting the mandibular nerve canal at intersection B. The length of the AB line was obtained by intersecting the mandibular nerve canal at intersection B. Six months after the operation, the patient’s preoperative and postoperative coronal panoramic radiographs were taken, and the distance measurements were read and recorded. Comparisons were made within and between groups. In order to achieve the smallest difference, all measurements were performed by one doctor.

### 2.6. Statistical Analysis

Each group’s mean and standard deviation were calculated. The paired *t*-test and Mann–Whitney U test were used to assess the difference in bone density before and after surgery for whether there was a statistically significant difference between groups. The *p*-values of <0.05 were considered to indicate statistical significance, and the analyses were performed using SPSS version 26.0 (SPSS Inc., Chicago, IL, USA).

## 3. Results

All 25 patients’ wounds healed within the first six months post-surgery, and all 36 surgical sites showed no inflammatory reactions such as redness, swelling, and collagen membrane exposure. Patient information and tooth extraction site information are indicated in Table 1. After six months post-surgery, CT scans showed that the height and the density of the alveolar bone had increased significantly.

Table 2 lists the alveolar dimensions at the maxillary extraction sites at the baseline and six months post-surgery. The alveolar height was increased by −2.15 ± 2.90 mm at the buccal crest; −2.45 ± 2.36 mm at the center of the socket; and −1.62 ± 3.19 mm at the palatal crest. Statistically significant differences were observed in the height of the buccal crest, as well as in the height in the center of the socket, before and after surgery (*p* < 0.05). No significant differences were estimated when comparing the height of the palatal crest before and after surgery.

The alveolar dimensions at the mandibular extraction sites at the baseline and six months post-surgery are listed in Table 3. The height of the buccal crest was increased by 0.19 ± 3.52 mm; and the height in the center of the socket was increased by −0.70 ± 2.71 mm. The height of the lingual crest was increased by −5.07 ± 4.34 mm, indicating a statistically significant difference before and after surgery (*p* < 0.05) (Figure 4a,b).

Table 4 shows the height of alveolar bone before and after bone grafting, by using the panoramic imaging technology of the oral surface layer. The *t*-test analysis of independent samples was compared: the preoperative bone height of the experimental group was 6.63 ± 3.75 mm; the bone height was 9.82 ± 3.72 mm at 6 months after the operation. There were significant differences between pre- and postoperative Alveolar dimensions (*p* < 0.001).

## 4. Case Report

**Figure 5:**
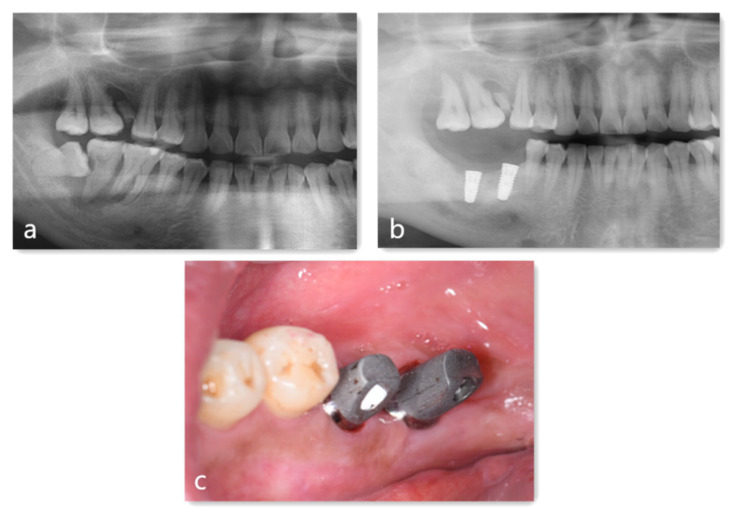
The treatment process of the case report. (**a**) Preoperative panoramic radiograph; (**b**) the panoramic radiograph 6 months after surgery; (**c**) Attaching abutments.

We referred a healthy 41-year-old male who presented to our hospital with a severely loose tooth at the right lower side. Clinical examination revealed that #46 was loosening to mobility (2), #47 was loosening to mobility (3), and #48 had slight pericoronitis. With the panoramic film showed in Figure 5a, the treatment plan was the replacement of #46 and #47 with implants and the extraction of #48.and GBR using AutoBT and Bio-Gide collagen membrane.

Subsequently, using extraction forceps and elevators if dispensable trauma to the surrounding alveolar bone walls should be avoided. The procedure required meticulous examination and careful debridement in order to remove all granulation tissue from the socket, followed by the use of sterile saline during irrigation and the need of fresh blood. AutoBT particles were made from teeth # 46 and # 47, and 5 mL of blood was drawn from the alveolar fossa and mixed with the AutoBT particles. The mixture was carefully placed into the bone defect area and covered with (Bio-Gide^®^) collagen. The details of the treatment process are shown in Figure 5a–c.

**Figure 6:**
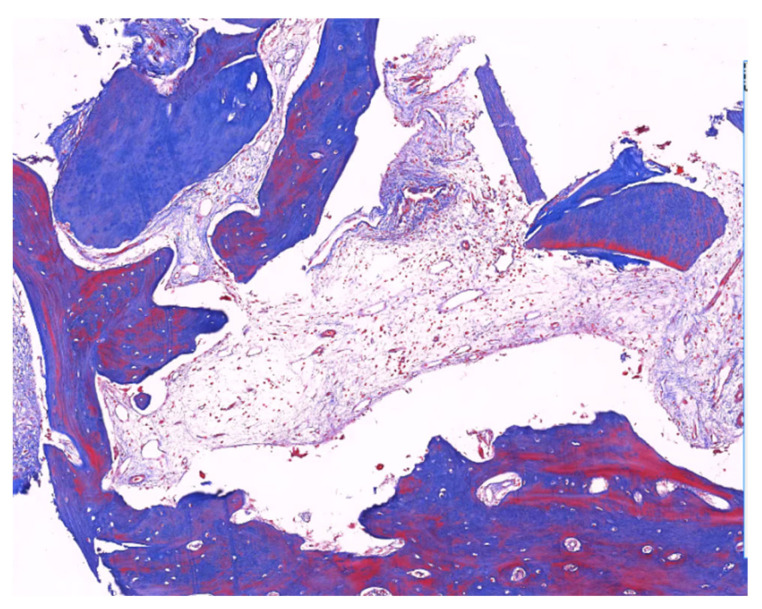
Histology of the grafted area for the AutoBT (H&E, magnification 200). The close contact of the newly formed bone with the bone substitute can be seen in the graft material.

The panoramic radiograph at 6 months showed that the AutoBT granules had been completely remodeled and fused with the original socket. Before the implant procedure, we took bone from the extraction socket. The collected specimens were immediately fixed with 10% formalin. Specimens were decalcified for 12 h and then processed by tap water rinsing and use of an automatic tissue processor (Supercentre XP, Shandon, Cheshire, UK). Paraffin-embedded sections were cut into 5 μm sections and examined under the microscope with hematoxylin-eosin staining (Figure 6).

All histological specimens were new bone, loose fibrous tissue, and abundant angiogenesis. AutoBT particles were completely mixed with the newly formed bone.

## 5. Discussion

This study may be the first trial to compare bone height before and after bone grafting on panoramic films and CBCT. It illustrates the effects on socket preservation of combining AutoBT with the severe periodontal patient’s extracted tooth. Six months post-surgery, a significant increase in bone volume and height was observed in the imaging at the maxillary and mandibular sites.

The results match previous studies, which stated that the collagen fibers in the dentin matrix were released after demineralization, along with some basic proteins, such as dentin sialoprotein and dentin matrix protein 1, from the platelet-rich plasma (PRP) [36,37]. The plasma fraction of autologous blood kept the platelet concentration above the baseline [38]. Plasma contains various key mitogenic and chemotactic growth factors [39]; these protein factors include BMPs, TNF-β, TGF-β, etc. [21,40], which may play an important role in bone healing [41].

Compared to a previous clinical study [18], this experiment has some shortcomings and lacks an assessment of the regeneration and retention of AutoBT in the alveolar ridge at the cytological level. In contrast, this experiment has a larger sample size compared to Minamizato [42] and uses visual radiological means in order to assess the effect of AutoBT in the alveolar crest. Additionally, (Bio-Gide^®^) collagen membranes, made from highly purified porcine collagen, were applied as a double-layered collagen membrane in this study. This membrane is commonly used in treating periodontal cases. The Bio-Gide^®^ collagen membrane is a promising alternative in guiding tissue regeneration, since it is slowly absorbable, potentially lasting for 9 months [43], and therefore has the potential to promote regeneration of the periodontal tissues. In this experiment, the surgeon covered the AutoBT with Bio-Gide^®^ collagen membrane in order to provide osteogenic space; however, the resorbable collagen membrane could not be used, as it lacked rigid support in order to maintain a stable osteogenic space. A case report by Gelețu GL [44] illustrates that pre-formed titanium mesh has some strength and rigidity to guide the regenerative bone contour and maintain a relatively stable space with good repair results.

AutoBT can be made into granules or blocks. According to the literature, some studies have shown that there is no significant difference in volume reduction between granular bone and block bone transplantation [45]. In this experiment, AutoBT was made into pellets, which were used to fill the alveolar sockets, but the presence or absence of resorption of the AutoBT pellets could not be determined because complete imaging of the marginal bone of the implants was not collected.

Several clinical [46] and experimental studies [47] have demonstrated the effectiveness of this membrane as a biological barrier in significant bone defects—either combined with a biomaterial, or used in isolation. This guided bone regeneration (GBR) technique is gradually being applied in clinical practice to effectively compensate for the lack of bone mass [48]. It provides a good cellular barrier, has a stabilizing effect on blood clots, and facilitates the binding of membranes to bone cells, thus enhancing osteogenesis and accelerating the healing of periodontitis. With socket preservation, alveolar bone absorption can be controled, thus providing sufficient bone for future implantation [49].

A histomorphometric study by Kang-Mi Pang in 2016, on specimens obtained after GBR using non-resorbable membrane and AutoBT granules, yielded the following result: new bone formation of AutoBT-grafted site was about 31.24% [42]. Histological studies showed that AutoBT was gradually absorbed and replaced by new bone around 6 months after transplantation [50]. In this study, the histological evaluation that was conducted 6 months after socket grafting showed favorable osteoconductive bone healing and remodeling as AutoBT particles started to be absorbed. A newly formed osteoid filled the absorbed area. Radiography showed the expected results in most cases. An X-ray performed 6 months after the bone graft revealed that the AutoBT particles showed a higher density of images around the bone graft area. It suggested that BOP and PI were perfect indexes by which to indicate peri-implantitis, so they might be added in further study [51].

Wound dehiscence has been reported in the literature as the most common complication in cases of site preservation using autogenous dental bone powder. The average wound dehiscence rate was 29.1% [45]. All of our surgical cases were performed by an experienced implantologist, and no cases of wound dehiscence have been observed.

CBCT is the use of a cone-shaped beam of radiation, which is picked up by a flat panel detector after the patient has been scanned [52]. The panoramic radiograph provides a complete image of the entire dental row and allows measurement of the bone density and quality of the maxilla and mandible [53]. In this study, CBCT and panoramic films of some patients were taken preoperatively and 6 months postoperatively.

If implants or metal crowns are already present in the patient’s mouth, their metallic component often results in a significant reduction in the resolution of the reconstructed CBCT images. This phenomenon is due to the high density of the metal, which blocks the rays and affects the ray data received by the detector, preventing the images of the metal areas from being processed properly, and creating artefacts [54]. These artefacts lead to a serious loss of image quality and can affect the doctor’s ability to measure the height of the alveolar bone. By fitting the pre- and postoperative CBCT with a 3D coordinate translation to a relatively fixed point, and using this as a basis for positioning of the implant site, the amount of postoperative bone height increase can be measured more accurately. However, the radiation dose of CBCT is 3–6 times higher than the radiation dose of the panoramic film [55], which is more suitable for follow-up because of its low radiation dose and favorable price.

Therefore, Planmeca panoramic radiographs and CBCT were selected in this study, in order to measure the changes in alveolar bone height in the patient before and after bone grafting, which indicates good authenticity and reliability. At the same time, it also reflects the clinically significant standard of care in evaluating the imaging selection of bone height.

There are several limitations to this trial. Firstly, the sample size was small and the follow-up period was relatively short. Secondly, the subsequent implant restoration and the survival of the implants were not followed up. Thirdly, the bone volume in the immediate post-surgical period was not accurately assessed.

## 6. Conclusions

Based on this 6-month clinico-radiological analysis, it seems that AutoBT can be used as an alternative bone material for preserving the alveoli after tooth extraction in patients with severe periodontitis, and it has a significant beneficial effect on bone augmentation on the buccal side of the extraction socket, as well as in the center of the cavity.

## Figures and Tables

**Table 1:** Demographic characteristics and clinical indices.

**Gender**	Male	11
	Female	14
**Age**	Mean	46
	Range	28–60
**Tooth position (N = 36)**	First premolar	3
	Second premolar	5
	First molar	14
	Second molar	14

**Table 2:** Alveolar dimensions at the maxillary extraction sites, baseline and 6 months post-surgery.

Parameter	Baseline	After 6 Mo.	Change	*p*-Value
BH, mm	3.25 ± 2.55	5.40 ± 2.74	−2.15 ± 2.90	0.001 **
CH, mm	4.50 ± 3.46	6.95 ± 2.63	−2.45 ± 2.36	0.001 **
PH, mm	3.33 ± 2.80	4.95 ± 2.51	−1.62 ± 3.19	0.053

Table key: BH: height of the buccal crest; CH: height in the center of the socket; PH: height of the palatal crest. ** *p* < 0.01.

**Table 3:** Alveolar dimensions at the mandibular extraction sites, baseline and 6 months post-surgery.

Parameter	Baseline	After 6 Mo.	Change	*p*-Value
BH, mm	11.86 ± 4.33	11.66 ± 2.59	0.19 ± 3.52	0.968
CH, mm	13.30 ± 2.665	14.01 ± 2.85	−0.70 ± 2.71	0.272
LH, mm	8.81 ± 4.58	13.88 ± 2.91	−5.07 ± 4.34	<0.001 ***

Table key: *** *p* < 0.001.

**Table 4:** Pre- and postoperative Alveolar dimensions and changes, as shown in the panorama.

Parameter	Baseline	After 6 Mo.	Change	*p*-Value
Bone height, mm	6.63 ± 3.75	9.82 ± 3.72	−3.19 ± 0.88	<0.001 ***

Table key: *** *p* < 0.001.

## Data Availability

Data are contained within the article. The data presented in this study are available in Table list.
